# Recent Advances in Genome-Editing Technology with CRISPR/Cas9 Variants and Stimuli-Responsive Targeting Approaches within Tumor Cells: A Future Perspective of Cancer Management

**DOI:** 10.3390/ijms24087052

**Published:** 2023-04-11

**Authors:** Khaled S. Allemailem, Saleh A. Almatroodi, Ahmad Almatroudi, Faris Alrumaihi, Waleed Al Abdulmonem, Wafa Abdullah I. Al-Megrin, Adel Nasser Aljamaan, Arshad Husain Rahmani, Amjad Ali Khan

**Affiliations:** 1 Department of Medical Laboratories, College of Applied Medical Sciences, Qassim University, Buraydah 51452, Saudi Arabia; 2 Department of Pathology, College of Medicine, Qassim University, Buraydah 51452, Saudi Arabia; 3 Department of Biology, College of Science, Princess Nourah bint Abdulrahman University, P.O. Box 84428, Riyadh 11671, Saudi Arabia; 4 Medical Supply Department, Durma Hospital, Durma 11923, Saudi Arabia; 5 Department of Basic Health Sciences, College of Applied Medical Sciences, Qassim University, Buraydah 51452, Saudi Arabia

**Keywords:** CRISPR/Cas9, Cas9 variants, gene expression, cancer therapy, tumor microenvironment, nanotechnology, stimuli-responsive nanoformulations, clinical trial

## Abstract

The innovative advances in transforming clustered regularly interspaced short palindromic repeats-associated protein 9 (CRISPR/Cas9) into different variants have taken the art of genome-editing specificity to new heights. Allosteric modulation of Cas9-targeting specificity by sgRNA sequence alterations and protospacer adjacent motif (PAM) modifications have been a good lesson to learn about specificity and activity scores in different Cas9 variants. Some of the high-fidelity Cas9 variants have been ranked as Sniper-Cas9, eSpCas9 (1.1), SpCas9-HF1, HypaCas9, xCas9, and evoCas9. However, the selection of an ideal Cas9 variant for a given target sequence remains a challenging task. A safe and efficient delivery system for the CRISPR/Cas9 complex at tumor target sites faces considerable challenges, and nanotechnology-based stimuli-responsive delivery approaches have significantly contributed to cancer management. Recent innovations in nanoformulation design, such as pH, glutathione (GSH), photo, thermal, and magnetic responsive systems, have modernized the art of CRISPR/Cas9 delivery approaches. These nanoformulations possess enhanced cellular internalization, endosomal membrane disruption/bypass, and controlled release. In this review, we aim to elaborate on different CRISPR/Cas9 variants and advances in stimuli-responsive nanoformulations for the specific delivery of this endonuclease system. Furthermore, the critical constraints of this endonuclease system on clinical translations towards the management of cancer and prospects are described.

## 1. Introduction

Cancer is a complex and multifaceted disease characterized by wide genomic instability, resulting in structural alterations that build up with tumor progression [1]. This disease is well characterized by mutations in genomic and epigenomic DNA that usually activate different oncogenes and suppress tumor suppressors. Cancer also dysregulates the epigenome, which coordinates normal gene expression regulation. This disease alters the normal cellular metabolism, cell structure, and motility and enables growth in inhospitable environments. Thus, a thorough knowledge of genomic changes, tumor microenvironment (TME), cellular adaptation, the defense system, and therapeutic response is crucial for developing effective treatment strategies against cancer [2].

A new era of genetic engineering started with the discovery of the clustered regularly interspaced short palindromic repeats-associated system (CRISPR/Cas), an adaptive immune system employed by bacteria and archaea against invading mobile genetic elements, plasmids, and bacteriophages [3]. The prokaryotes acquire short genomic segments from such invaders and integrate them within their genetic code; this serves as a molecular memory during any subsequent infection from the same infectious elements [4]. These acquired sequences are further transcribed as a part of the CRISPR array to form CRISPR RNA (crRNA). The crRNA serves as a guide for Cas endonuclease to recognize any infectious genetic material that matches the previous genetic target [5]. The endonuclease activity of Cas is also determined by the protospacer adjacent motif (PAM), a 2–6 bp nucleotide sequence that serves as a double check to distinguish Cas from the foreign genetic material before its degradation.

The recent advances in the CRISPR/Cas9 system have gained tremendous attention for their precise genome targeting and editing in different model systems, including human cells. This system has been repurposed as a robust tool for RNA-guided DNA targeting for genome editing. In addition to genome editing, this system has been applied for transcriptional regulation, epigenetic modeling, and genome imaging. CRISPR/Cas9-based genome editing has emerged as a versatile tool for the study and treatment of diverse cancers [6]. With the help of the CRISPR/Cas9 system, precise manipulation of any DNA sequence is possible, defined by a short stretch of guide RNA (gRNA) [7]. This technique allows us to elucidate the proper role of genes in the development of different diseases and their progression.

The currently most widely used CRISPR/Cas9 system is SpCas9, which possesses robust DNA targeting and cleavage activity. It contains 1368 amino acid residues, can be used with either a crRNA/trans-activating CRISPR RNA (tracrRNA) pair or sgRNA, recognizes a relatively common NGG PAM, functions optimally with 20-nt spacers, and supports relatively high levels of off-target editing [8]. However, some Cas9 variants possess more advantages over SpCas9, such as SaCas9, which is a smaller-sized endonuclease with 1,053 amino acid residues [9] and requires pyrimidine-rich PAMs (for example, Nme2Cas9) [10].

*Streptococcus pyogenes* Cas9 (SpCas9) has been extensively used as a genome editing tool, owing to its relatively broad PAM compatibility and high activity. Since the initial reports about the in vitro and mammalian cell-programmed DNA cleavage by SpCas9 nuclease [11], different Cas9 orthologs from *Streptococcus thermophiles* [12], *Staphylococcus aureus* [9], *Campylobacter jejuni* [13], *Neisseria meningitides* [10], and many other organisms [14] have been discovered. These Cas9 effectors differ in their overall size, gRNA architecture, PAM sequence, editing specificity/efficiency, and optimal spacer length.

Applications of SpCas9 are sometimes compromised by its off-target effects or lack of its PAM sequence (NGG), which limits further use. To overcome all these limitations, SpCas9 variants have been engineered, and some of the high-fidelity variants include SpCas9-HF1 [15], eSpCas9 (1.1) [16], evoCas9 [17], HypaCas9 [18], and Sniper-Cas9 [19]. The variants with broadened or altered PAM compatibilities comprise QQR1 [20], VQR [21], VRQR [15], VRER [21], as well as SpCas9-NG [22], and xCas9 [23]. However, some variants like xCas9 and VRQR-HF1 presented broadened or altered PAM compatibilities as well as enhanced fidelity [15,23].

In addition to sequence alteration, Cas endonucleases have been recently modified in different ways to broaden their action at target locations. Some of the modifications include the fusion of adapter proteins, such as transcriptional activators/repressors, deaminases, and reverse transcriptases [24]. With these modifications, the CRISPR/Cas system has opened a new setup in genome-editing technology, disease management, and therapeutic strategies.

The proper delivery of the CRISPR/Cas9 genome-editing system within target cells is also a great challenge. Nanotechnology significantly contributes to the nanoformulation design, transport, and payload delivery of different agents and anticancer drugs within the target sites. Nanotechnology-based delivery of CRISPR/Cas9 for genome editing within tumor cells paves the way for its clinical translation. However, different barriers still exist for the proper and safe delivery of CRISPR/Cas9 nanoformulation, which needs to be sorted out in the near future.

In this review, we aim to elaborate on different CRISPR/Cas9 variants and their regulation with different small molecules and proteins to make the surrounding environment responsive. The advances in stimuli-responsive nanoformulations for the specific delivery of this endonuclease system are described. Furthermore, some critical constraints of the CRISPR/Cas9 system for clinical translations toward the management of cancer and future prospects are discussed.

## 2. Different Cancer Treatment Approaches and Their Limitations

Although significant development has been achieved in medicine, cancer is still known as the most dreadful disease, as millions of people die globally due to this disease. Tremendous efforts are put forward to look for more unique therapeutics to overcome the limitations of conventional treatment methods. In the recent past, some other strategies have been introduced as cancer treatment approaches. Some distinguished strategies for this disease management include the use of natural antioxidants, extracellular vesicles (EVs), gene therapy, targeted therapy, radiomics, pathomics, and thermal ablation [25].

Nanomedicine in the form of nanoparticles (NPs) has been recently used as conventional chemotherapeutic drugs as a platform for biocompatible and biodegradable systems. This system enhances the bioavailability of chemotherapeutics near the cancerous mass. However, the engineering of such nanoformulations with anticancer drugs—specific to each cancer type, loading capacity, and targeted delivery—is a big challenge that needs to be sorted out further [26].

Circulating EVs aid in the early identification of cancer biomarkers, which can be isolated to exploit as anticancer vaccines for tumor therapy [27]. However, isolation, quantification, and drug loading are major challenges related to the use of EVs.

The techniques of thermal ablation and magnetic hyperthermia for different tumor masses have the advantage of localized treatment in narrow and precise areas. However, some limitations, like its application in localized areas only, low penetration power, and the need for highly skilled specialists, limit its use [28].

Some recent modes of cancer management, such as radiomics and pathomics, significantly contribute to data collection for other treatment approach applications [29,30]. However, these strategies are difficult and laborious, as these approaches require procedure standardization and statistical/computational methods to be set up to facilitate clinical translation.

Another promising cancer treatment approach lies in gene therapy through targeted silencing by small interfering RNA (siRNA) for the possible expression of apoptosis-triggering genes and wild-type cancer suppressors [31]. However, several challenges remain that limit the application of gene therapy. These challenges include smart delivery approaches of RNAi, high neutralization chances by the immune system, controlled RNA interference, and limited efficacy in specific cancer patients [32].

Transcription activator-like effector nucleases (TALENs) and zinc-finger nucleases (ZFNs) are powerful tools, redefining the boundaries of biological research. However, these nucleases also face some limitations, like the target-based design of TALENs and ZFNs, which limit their broad applications for cancer treatment. These nucleases have been used to modulate some oncogenes, rendering them non-functional [33].

Compared to all these cancer treatment procedures, the approach using CRISPR/Cas9 is considered the most innovative and excellent, offering several advantages. These advantages include high efficacy, target design simplicity, and application for inducing multiple mutations. CRISPR/Cas9 offers great promise for the identification of essential genes that regulate different biological activities. In addition, this genome-editing tool offers significant promise in drug targeting against a wide range of diseases. Furthermore, CRISPR/Cas9 is used for the induction of DNA modification by CRISPR activation (CRISPRa) or CRISPR interference (CRISPRi) [34]. However, there are still some limitations with this system, such as off-target effects, which need to be sorted out in the near future.

## 3. CRISPR/Cas9 Structure and Mechanistic Action

**Figure 1:**
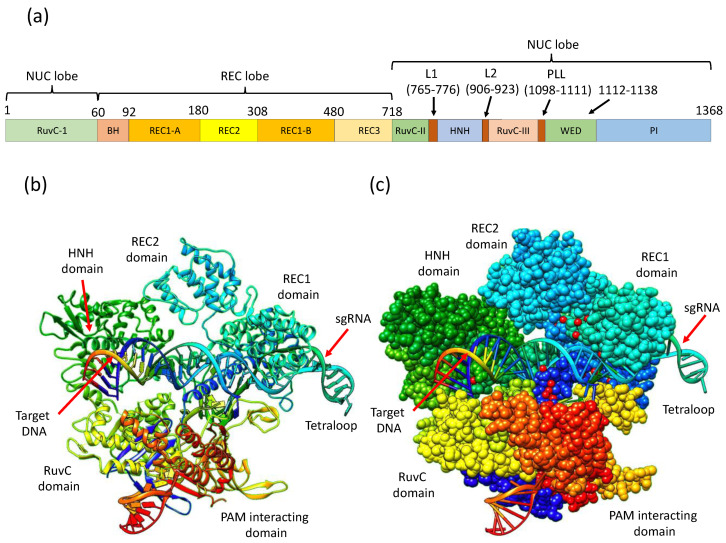
Domain organization of SpCas9 and three-dimensional structure of SpCas9-sgRNA-DNA ternary complex shown by (**a**) domain organization of Cas9, (**b**) ribbon representation, and (**c**) space-filling model, obtained from protein data bank (PDB) at https://www.rcsb.org (accessed on 12 January 2023), PDB ID: 4OO8 and edited by UCSF Chimera.

The crystal structure of full-length *S. pyogenes* Cas9 at 2.5 A° resolution in complex with 98 nt sgRNA and 23 nt target DNA with 1368 residues has been resolved with the single wavelength anomalous dispersion (SAD) method by using seMet-labeled protein technique [35]. This CRISPR/Cas9 complex has a bilobed architecture with target recognition (REC) and nuclease (NUC) lobes. These lobes possess a positively charged groove at their interface to accommodate sgRNA:DNA heteroduplexes. The NUC lobe contains HNH and RuvC domains, whereas the REC lobe binds sgRNA and DNA heteroduplex [6]. Cas9 endonuclease forms a complex with either CRISPR RNA (crRNA) or trans-activating crRNA (tracrRNA). However, crRNA and tracrRNA can be engineered to form a single RNA complex called single-guide RNA (sgRNA) that forms a complex with the Cas9 enzyme and targets a complementary genetic sequence for cleavage (Figure 1).

**Figure 2:**
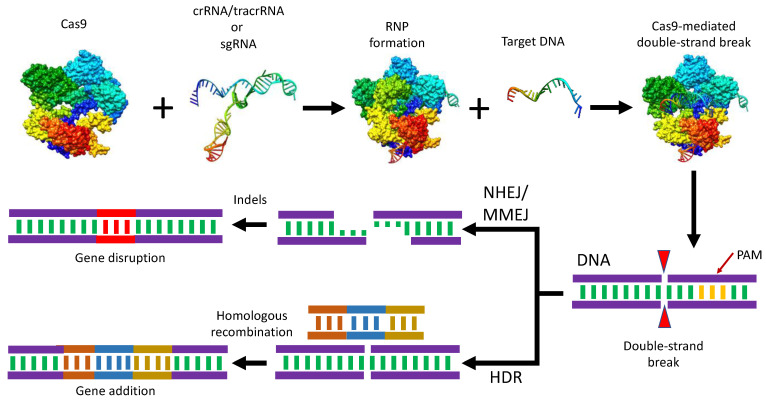
Multiple genomic modifications followed by cleavage of target DNA by CRISPR/Cas9. At DNA breaks, due to some mistakes in DNA repair by the endogenous NHEJ or MMEJ pathways, variable-length insertions and/or deletions (indels) can be formed. Alternatively, a DNA repair template created through the HDR pathway creates defined insertions, deletions, or other specific modifications.

A short ∼20 bp nucleotide sequence present in crRNA recognizes the target sequence. In addition, a short sequence (5′-NGG-3′) known as the protospacer adjacent motif (PAM) is required very near the downstream of the target sequence for Cas9-mediated cleavage [36]. Directly upstream of the PAM, between the third and fourth nucleotides, the cleavage of the phosphodiester bond of the target DNA takes place, resulting in a blunt-end double-strand break (DSB) [37]. In eukaryotes, DSB repair mechanisms involve either homology-directed repair (HDR), nonhomologous end joining (NHEJ), or microhomology-mediated end joining (MMEJ), also known as an alternative to NHEJ [38]. The HDR is a more precise editing mechanism as compared to the NHEJ repair pathway. The HDR repair strategy requires a homologous DNA sequence as a repair template [39]. HDR mechanisms can be used to knock in some exogenous donor sequences within the target DNA. However, the NHEJ pathway is error-prone; it requires no template, and the ligation of two nascently cleaved DNA strands often occurs with the addition or deletion of adjacent nucleotides. This usually results in insertion-deletion (indel) mutations that result in frameshift mutations, resulting in the knock-out (KO) of genes (Figure 2).

A key limitation of SpCas9 is the strict requirement of NGG PAM at the target site. This restriction remains a check for broad genome editing applications that require precise Cas9 positioning. Recently, new Cas9 variants have been engineered with mutations in the RuvC or HNH domains, and such Cas9 variants exhibit only nickase activity [39]. Furthermore, such Cas9 variants can be further fused with reverse transcriptase for prime editing, and deaminase fusion with these variants results in adenine/cytidine base editing [38]. A completely inactive form of Cas9, dead Cas9 (dCas9), can be engineered by introducing mutations in both nuclease domains. This variant of Cas9 (dCas9) can be fused with other effector proteins, such as transcriptional repressors or activators, to achieve programmable RNA-guided epigenetic regulation [40].

Although SpCas9 is the most popular nuclease, the search for other naturally occurring alternatives to Cas9 forms is still going on. The different alternatives to Cas9 are Cas3, CasX, CasY, Cas12a, Cas12b, Cas13a, Cas13b, Cas13d, and Cas14a [41]. If we broadly check the major differences between Cas9/Cas9 variants and other Cas9 alternatives, for example, Cas12a (previously known as Cpf1), it is reported that Cas9 requires two RNA molecules to cut the target DNA, while Cas12a needs only one. Cas12a lacks tracrRNA, uses a T-rich PAM, and cleaves DNA via a staggered DNA DSB [42]. Cas9 cuts both the target DNA strands at the same place, while Cas12a cuts them at different positions. Thus, Cas9/Cas9 variants leave behind blunt ends, while Cas12 leaves behind one strand shorter than the other, creating sticky ends. Compared to blunt ends, sticky ends possess some different properties during NHEJ or HDR repair of DNA. This difference confers certain advantages to Cas12a as compared to Cas9/Cas9 variants when attempting gene insertions [43]. Target genes can be efficiently disabled by CRISPR/Cas9/Cas9 variants; however, it is challenging to generate a knock-in or insert the gene in target DNA.

## 4. Cas9 Engineering to Generate Its New Variants

As we know, bacteria and archaea have adopted CRISPR/Cas9-mediated genome-editing strategies as an adaptive immune system against mobile genetic elements, including plasmids and viruses [44]. Phages can, however, smartly induce mutations in the target genome to escape this bacterial immune surveillance [45]. To counteract this strategy, the native CRISPR/Cas9 system has evolved to adapt the mismatch tolerance by bearing some nucleotide mismatches, e.g., in the PAM region, thus impeding viral immune evasion [23].

Compared to the bacterial genome size, the mammalian genome size is thousands of times larger, thus there are far more chances of off-target occasions when the CRISPR/Cas9 system is used to edit the mammalian genome [46]. However, the recent progress in the use of different high-fidelity variants of Cas9 suggests that the protein engineering of this endonuclease system can overcome some off-target limitations.

Different strategies have been used to produce new Cas9 variants showing high fidelity rates. These strategies are generally classified as rational, nonrational, or combined methods. A rational approach involves structural and/or functional knowledge, especially through computational modeling or point mutations to generate new Cas9 variant designs [16]. Nonrational tactics are established on the basis of an evolution-based strategy, typically involving random mutagenesis followed by high-throughput screening [47]. The combined strategy to generate new Cas9 variants involves direct evolution and structure-guided engineering.

The amino acid substitution in Cas9 led to the design of different high-fidelity Cas9 variants, viz., evoCas9 [48], eSpCas9 [49], HypaCas9 [50], SpCas9-HF1 [51], Sniper-Cas9 [52], SpCas92Pro [53], and xCas9 3.7 [54]. The Cas9-based toolbox has been modified to accommodate catalytically impaired, nuclease-dead Cas9 (dCas9), or Cas9 nickase forms [55]. This form of Cas9 does not cleave DNA, but under the guidance of specific sgRNA, it can bind DNA precisely and specifically [56]. dCas9 is used to recruit different repressors or transcriptional activators for targeted gene repression or activation. The recruitment of epigenetic modifiers at specific genomic locations to achieve specific epigenetic modifications is also performed by using dCas9.

Due to its high activity in eukaryotic cells and short PAM requirement, SpCas9 is the most studied CRISPR/Cas9 nuclease type. Different Cas9 variants, mutation location, year of first introduction, and different cell types studied in relation to each variant is presented in Table 1.

Some well-known variants of SpCas9 and their important properties are described below:

### 4.1. SpCas9 Nickase

**Figure 3:**
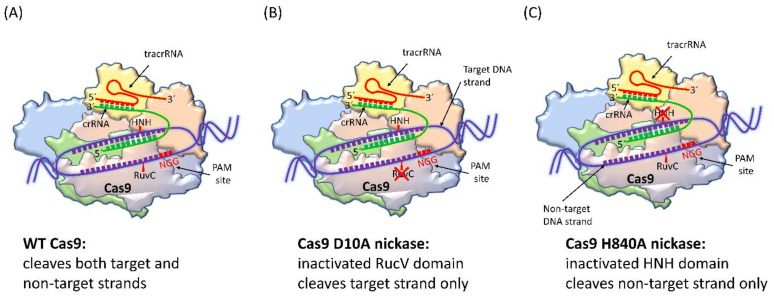
Generation of target strand and non-target strand SSBs by Cas9 nickase. (**A**) WT Cas9 possesses active HNH and RuvC domains, which cleave both the strands of target DNA, while (**B**) Cas9 D10A nickase cleaves only target strand DNA due to its inactive RuvC domain, and (**C**) Cas9 H840A nickase cleaves only non-target strand due to its inactive HNH domain.

Due to low stringency for DNA complementarity in wild-type (WT) SpCas9, Cas9 nickases have been engineered to create new variants. These variants create single-stranded breaks (SSBs) rather than double-strand breaks (DSBs). SpCas9 nickase includes a D10A point mutation that produces RuvC nuclease domain inactivation; thus, this form of nickase cleaves only the target DNA [68]. For the generation of DSB, two adjacent gRNAs are used with paired nickases. Thus, the possibility of off-target effects is effectively excluded, as both Cas9 nickases must nick their targets for the generation of a DSB (Figure 3).

The advantage of using two Cas9 nickases is that it leads to the formation of cohesive ends with greater control over gene insertion and integration. This special quality of CRISPR nickases makes it an ideal genome editing system for therapeutic applications. The genome editing strategy in primary T cells usually involves nickases, in addition to the excision of viral DNA (e.g., HBV) in humans [69]. For nickase applications, different DNA strands must be targeted by gRNAs to create a DSB. This can be accomplished with either a PAM-in or PAM-out orientation. PAM-in designs place the PAMs closer together in the middle of the targeted region, whereas PAM-out designs have the PAM sequences on the extremes of the targeted region.

The ovarian cancer cells (SKOV3 and OVCAR3) highly express survival, which strongly correlates with the patient’s overall poor survival. Survivin is highly expressed in different cancers as compared to normal tissues. This cancer marker protein plays a significant role in epithelial-to-mesenchymal transition (EMT). The lentiviral CRISPR/Cas9 nickase in these cancer cells led to BIRC5 gene editing, which resulted in the inhibition of EMT by upregulating epithelial cell markers and downregulating mesenchymal markers in both ovarian cancer cells [70].

### 4.2. dCas9-FokI

**Figure 4:**
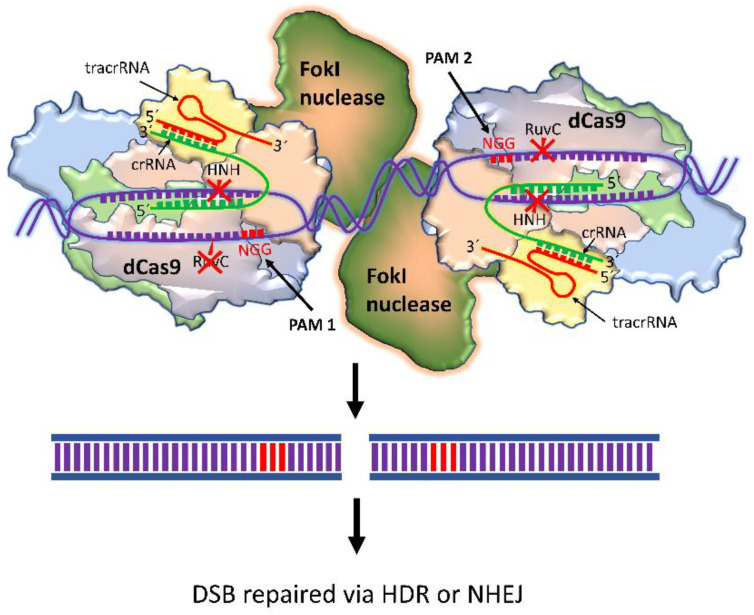
DNA cleavage by dCas9-FokI nuclease. FokI dimerization generates a double-stranded break at a specific sequence.

The restriction endonuclease *Fok*1 is naturally found in *Flavobacterium okeanokoites*. dCas9-FokI is composed of dead Cas9 (dCas9) fused with the FokI endonuclease. As FokI cleaves a DNA only after dimerization, dCas9-FokI works in pairs. The binding affinity of dCas9 with gRNA and target DNA is not affected by its deactivation. dCas9 is guided by gRNA to the target site, and FokI performs the cleavage. When FokI dimerizes, it makes a DSB at a specific target sequence. It requires two unique gRNAs that bind 15–25 bp apart for dCas9-FokI to dimerize at a target site [60]. This strategy reduces the unwanted off-target effects of CRISPR, like nickase technology (Figure 4).

Several artificial transcription factors (ATFs) have been used in combination with CRISPR/dCas9 in cancer therapy. This system manipulates the DNA to modify some target genes, silence oncogenes, activate some tumor suppressor genes, and silence tumor resistance mechanisms for targeted therapy. In addition, the use of CRISPR/dCas9-based ATFs in combination with drug repurposing could be an alternative cancer treatment strategy [71].

### 4.3. SpCas9-D1135E

Kleinstiver et al. (2015) accidentally observed that, as compared to WT SpCas9, engineered SpCas9 nucleases possessing altered PAM specificities (the D1135E mutant) could yield a widespread enhancement in genome-wide specificity. This has been evidenced by GUIDE-seq experiments, and this was the first observation showing that point mutations can enhance the genome-wide specificity of SpCas9 [21]. A recent novel study identified that at noncanonical NAG/NGA PAM sites, the D1135E variant exhibits much reduced editing activity, while this variant at canonical NGG-flanking sites preserves robust on-target activity [72]. The role of this Cas9 variant has not been explored in any type of cancer yet.

### 4.4. eSpCas9

Enhanced specificity Cas9 (eSpCas9) represents a variant that is a structure-guided protein engineered from WT SpCas9. Slaymaker et al. (2016) observed that when the strength of Cas9 binding to the non-target strand exceeds that of DNA rehybridization, off-target cleavage occurs [16]. Later, the researchers proposed that this model maintains robust on-target cleavage and reduces off-target effects. The eSpCas9 variant is used for genome-editing applications that require a higher level of specificity. The research group identified three mutants with both high efficiency and specificity: SpCas9 (K855A), SpCas9 (K810A/K1003A/R1060A) [also referred to as eSpCas9 (1.0)], and SpCas9 (K848A/K1003A/R1060A) [also referred to as eSpCas9 (1.1)]. These three variants broadly retained efficient nuclease activity, measuring on-target indel generation at 24 target sites spanning 10 genomic loci [16]. In human cells, eSpCas9 (1.1) and high-fidelity Cas9 (SpCas9-HF1) variants display significantly reduced off-target cleavage, but the mechanism of this target discrimination is unknown [15].

### 4.5. SpCas9-HF1 (-HF2, -HF3, -HF4)

SpCas9-HF1 represents a high-fidelity variant of WT SpCas9 that harbors alterations designed to minimize non-specific DNA contacts. This variant holds on-target events similar to WT SpCas9 with >85% of sgRNA tried in human cells. Nearly no off-target events were detectable by SpCas9-HF1 targeted to standard non-repetitive sequences, checked by target sequencing and genome-wide break methods. Even for atypical, repetitive target locations, the major off-target mutations promoted by WT SpCas9 were undetected with SpCas9-HF1. Kleinstiver et al. further induced SpCas9-HF1 with other point mutations to generate SpCas9-HF2 (HF1 + D1135E), -HF3 (HF1 + L169A), and -HF4 (HF1 + Y450A). It has been observed that these new variants of SpCas9-HF1 could further minimize indel frequencies at some off-target locations that remain for SpCas9-HF1. With its outstanding precision, SpCas9-HF1 offers an alternative to WT SpCas9 for therapeutic applications and research purposes [21,57].

### 4.6. HypaCas9

It has been reported that in human cells, eSpCas9 (1.1) and SpCas9-HF1 variants show considerably reduced off-target cleavage. The targets of discrimination and improvement in fidelity are unknown [15,16]. Thus, using a single-molecule Forster resonance energy transfer (FRET) experiment, Doudna’s research group showed that when bound to mismatched targets, both eSpCas9 (1.1) and SpCas9-HF1 are trapped in an inactive state [73]. It was also observed that REC3 and the non-catalytic domain within Cas9 recognize target complementarity and rule the HNH nuclease to control the general catalytic competence. By exploiting this phenomenon, the same research group engineered a new, hyper-accurate Cas9 variant (HypaCas9) that exhibits, in human cells, high genome-wide specificity without conceding on-target activity.

This variant has also recently been reported to accurately edit mouse zygotes. This variant proficiently modified the target locus even in a single-nucleotide mismatched sequence. With the application of HpCas9 for the discrimination of single nucleotide polymorphisms (SNPs) in hybrid strain-derived zygotes, allele-specific gene modifications and the generation of monoallelic mutated mice were accomplished. The results suggested that the improved accuracy of HypaCas9 could facilitate the genetic modification of animals [63].

### 4.7. HiFi Cas9

For the modification of stem cells with CRISPR/Cas9, specificity remains a major concern. It has been observed that with the engineered Cas9, the therapeutic application of the high-fidelity variant shows reduced on-target activity when used by the ribonucleoprotein (RNP) delivery method. To sort out this issue, a bacterial screening method was devised to isolate the variants that retain higher activity in the RNP format. It was observed that a single point mutation, R691A, known as high-fidelity (Hifi Cas9), retained improved on-target activity and reduced off-target editing. HEK293 cells were used for targeting the 12 sites within the HPRT locus by five Cas9 RNPs, including WT SpCas9, Hifi Cas9, SpCas9-HF1, eSPCas9 (1.1), and HypaCas9. The median on-target activity determined by next-generation sequencing (NGS) for each variant was observed to be 82% for Hifi Cas9, 2% for SpCas9 HF1, 20% for eSpCas9 (1.1), and 1.7% for HypaCas9 [64]. It is presumed that, for the sake of enhanced editing specificity, the multipoint mutagenic high-fidelity SpCas9 mutants are overengineered, and their relatively low on-target activity as compared to WT SpCas9 might be covered up when delivered as overexpression plasmids [64].

### 4.8. xCas9

Phage-assisted continuous evolution was used to evolve SpCas9, and this led to the generation of xCas9 variants (−3.6 and −3.7) having expanded PAM compatibility [23]. xCas9 3.7 demonstrates higher DNA targeting and broad PAM compatibility when complexed with sgRNA and double-stranded DNA targets. Structural comparisons have demonstrated that salt bridge-stabilized R1335 is crucial for the rigorous selection of the PAM sequence by SpCas9 [74]. However, the unrestricted rotamerization of this residue by the E1219V mutation in xCas9 3.7 minimizes the PAM recognition strictness and allows SpCas9 to identify multiple PAM sequences. xCas9 3.7 REC2 and REC3 domains undergo prominent conformational changes as compared to WT SpCas9. These changes lead to reduced contact with the DNA substrate. The xCas9 3.7 variant displays less interaction with DNA and possesses more flexible REC2 and REC3 domains with enhanced specificity for a DNA substrate. The broadened PAM compatibility in xCas9 3.7 can assist rational engineering for more efficient SpCas9 variants and other Cas9 orthologs.

A broad PAM compatibility including NGG, NGA, and NGT has been reported with xCas9 (3.7) in both embryos and founder rabbits. Precise gene modification could be performed with optimized xCas9, which has enhanced base editing efficiency [75].

### 4.9. Sniper-Cas9

Without attenuating the cleavage activity of SpCas9, Lee et al. utilized the directed evolution method in *E. coli* to improve the specificity of this endonuclease system [19]. New SpCas9 variants having higher activity and specificity were isolated by sniper screening. Simultaneous positive and negative selection of SpCas9 variants is allowed by this screening without killing the on-target activity. Sniper-Cas9 exhibited many inferior off-target effects than the WT at all sites, and off-target sites were not cleaved when compared with SpCas9 [19]. Sniper-Cas9 presented superior on-target activities in comparison with the engineered high-fidelity SpCas9 variants such as HypaCas9, SpCas9-HF1, eSpCas9 (1.1), and evoCas9. However, stronger tolerance to single mismatch at the PAM-distal region was exhibited by Sniper-Cas9 [19].

### 4.10. evoCas9

For therapeutic purposes specifically, the potential for off-target activity of WT CRISPR/Cas9 limits their applications [76,77]. Casini et al. developed a yeast-based procedure for the identification of optimized SpCas9 variants that simultaneously enable evaluation of on- and off-target activities [17]. The research group screened the SpCas9 variant library with random mutations in the REC3 domain and identified mutations that enhanced the editing accuracy while maintaining the editing efficiency. Four beneficial mutations were combined to generate the new variant called evoCas9. This variant possesses fidelity exceeding both wild-types (79-fold improvement) and rationally engineered Cas9 variants (fourfold average improvement) [15,16]. evoCas9 variant maintained nearly WT on-target efficiency (90% median residue activity). The evoCas9 variant presented significantly enhanced specificity at endogenous genomic loci, and no off-target sites were observed for four of the eight sgRNAs tested. Furthermore, after long-term exposure of 40 days, evoCas9 strongly limited the nonspecific cleavage of difficult-to-distinguish off-target sites and entirely abolished the cleavage of two-dimensional off-target sites.

### 4.11. SpartaCas9

A mutagenesis method named scanning mutagenesis of oligo-directed targets (SMOOT) was developed as an evolutionary strategy to screen SpCas9 variants, retaining efficient on-target activities with low off-target editing [65]. Highly distinct libraries of SpCas9 variants followed by high-throughput M13 bacteriophage-mediated selection were selected. This led to the discovery of a new mutant, termed *S. pyogenes* Adopted to Reduce Target Ambiguity Cas9 (SpartaCas9), comprised of the majority of supplemented point mutations. This variant was observed to have high on-target editing in T-cells with minimal off-target effects [65].

### 4.12. LZ3 Cas9

The analysis of double-strand cleavage events has been studied by tagmentation-based tag integration site sequencing (TTISS), the technique developed by Schmid-Burgk et al. [61]. By using this technique, the researchers compared WT SpCas9 and eight high-fidelity SpCas9 variants. It was revealed that there is a tradeoff between activity and cleavage specificity. A saturation mutagenesis of 157 residues present in RuvC and HNH domains and LI and L2 linkers has been carried out to evaluate whether this tradeoff is a general feature. The research group further combined the top point mutations exhibiting high specificity and on-target efficiency to present the combinatorial mutants. This led to the identification of an LZ3 Cas9 variant that presented enhanced specificity and high on-target activity relative to the WT Cas9 [61].

### 4.13. miCas9

MiCas9 comprises a fused minimal motif consisting of thirty-six amino acid residues that are added to SpCas9 to improve its HDR repair capacity [66]. Through the fusion motif, MiCas9 binds with RAD51 and enriches it at the target site. MiCas9 augments the large-size gene knock-in rate in comparison to WT SpCas9, thus systematically reducing the off-target insertion and deletion incidents. This approach also increases the single-stranded oligonucleotide-mediated specific genome editing rate and efficiently minimizes on-target insertion and deletion rates in knock-in situations. Thus, MiCas9 has a broad application in genome editing research and therapeutics in the future [66].

### 4.14. SuperFi-Cas9

While looking for the different variants of Cas9, the target selectivity results either in increased-fidelity nuclease (IFN) at some targets, while at others it translates into either fully or partially reduced activity [62,78]. A newly engineered variant was developed in recent research that can go beyond this paradigm [79]. This variant was engineered by exploiting the rational design of SpCas9 in cryo-electron microscopy structures with PAM distal mismatching sgRNA. The new structure showed that the distorted end of the target DNA-sgRNA hybrid helix is stabilized by a flexible loop of the RuvC domain. This allows SpCas9 activation even in the presence of various mismatches. The residues that stabilize the distorted helix end were not involved in any interactions in any known SpCas9 complexed with on-target DNA. Furthermore, the off-target cleavage activity of SpCas9 could be minimized without affecting the on-target cleavage by disrupting these mismatch-stabilizing interactions.

Based on this approach, a new enhanced-fidelity SpCas9 variant was engineered by introducing mutations at seven contacting residues to aspartic acid. By using an in vitro target/off-target pair, this new variant exhibited WT-like on-target activity and decreased off-target activity. It was based on SpCas9-HF1 and Hypa-SpCas9 and two IFNs, previously reported to be two orders below WT. This new generation variant was named SuperFi-Cas9, inspired by its dual on-target activity and high-fidelity potential [67].

## 5. Comparison between Different Cas9 Variants

The most common cause of alteration in different Cas9 variants is the amino acid substitution of critical domains. Although each variant exhibits greater target specificity, each variant has some limitations. These limitations include low activity, limited scope, and strict PAM dependence. Thus, further studies need to be conducted to enhance the genome editing efficiency of each variant. An optimal variant needs to be selected for a given target based on activity comparison, PAM compatibility, and specificity. The mechanism and important characteristics of each Cas9 variant are summarized in Table 2.

By comparing different SpCas9 variants, the results presented showed that the overall activity order of high-fidelity variants could be graded as SpCas9 ≥ Sniper-Cas9 > eSpCas9 (1.1) > SpCas9-HF1 > HypaCas9 ≈ xCas9 > eVoCas9. The overall specificity could be ordered as evoCas9 > HypaCas9 ≥ SpCas9-HF1 ≈ eSpCas9 (1.1) > xCas9 > Sniper Cas9 > SpCas9 [62].

**Figure 5:**
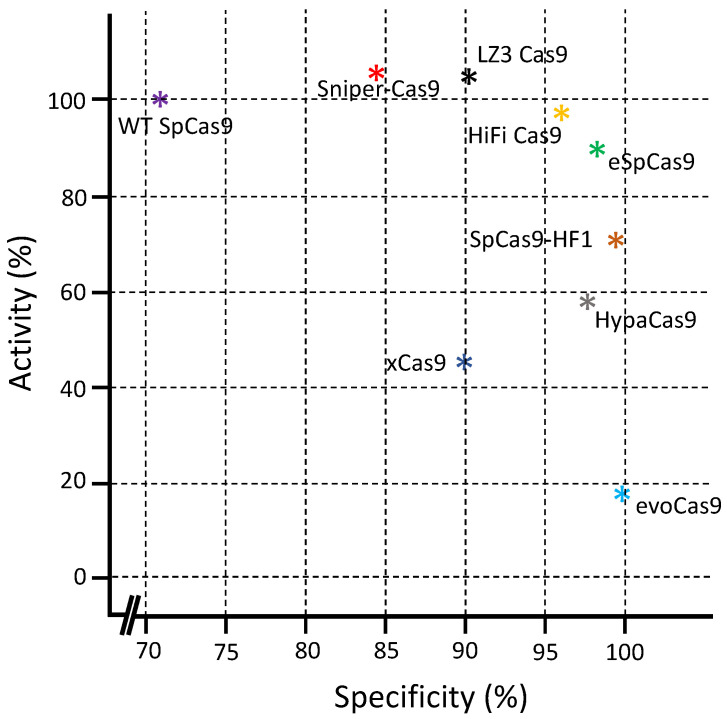
Specificity and activity scores for some tested SpCas9 variants [61].

In addition, for the comparison of specificity and activity between different Cas9 variants, Schmid-Burgk et al. calculated the scores between these two parameters. By the measurement of on-target indel frequencies by targeted sequencing, it was revealed that xCas9 (3.7) and evoCas9 possessed the lowest on-target activity, while HiFi Cas9, LZ3 Cas9, and Sniper-Cas9 possess WT Cas9 comparable on-target activity [61] (Figure 5).

## 6. Allosteric Modulation of Cas9-Targeting Specificity

Cas9 is a typical allosteric enzyme that undergoes a series of clear-cut conformational rearrangements from the target recognition state to the cleavage level [81,82]. This progression encompasses multiple layers of fine allosteric regulation to ensure its precise functional activity and target precision [55]. The structure of native Cas9 in different stages includes the apo-state [83], sgRNA-bound pre-targeting state [83], incomplete DNA-bound intermediate state [84], dsDNA-bound pre-cleavage state [85], R-loop-bound pseudo-active state [86], cleavage state [87], post-cleavage state [88], and product state [88]. A knowledge of all these states is helpful to understand the allosteric effects and conformational transition pathway along which native Cas9 succeeds in its nucleation activation.

The Cas9 HNH domain undergoes a significant transition and rotation towards the cleavage state upon target DNA binding, and during this state, a conformational checkpoint has been identified that determines whether Cas9 cuts its bound target DNA [89]. The HNH domain tends to be trapped in this checkpoint intermediate if the number of gRNA-target DNA mismatches at the PAM-distal end exceeds a threshold. These studies have been confirmed by single-molecule Forster resonance energy transfer (FRET) reports [89].

A hyper-accurate Cas9 variant (HypaCas9) was engineered from the observation of this unique allosteric control in Cas9. Two amino acid mutations were introduced in the PAM-distal REC3 domain to elevate the energy barrier underlying the HNH domain reorientation [50]. A remarkable folding-unfolding transition occurs in two linker regions (L1 and L2) that connect the HNH and RuvC nuclease domains upon dsDNA binding [67]. These two linkers can work as allosteric transducers to mediate definite cleavage of both DNA strands [82].

The molecular dynamics (MD) simulation has been used to study the allosteric crosstalk between the Rec lobe, HNH, and RuvC domains to ease the assembly of the Cas9 effector complex [90]. Furthermore, Mg^2+^ performs its essential role in activating the Cas9 conformations in addition to its role in catalysis. The FRET reports reveal that the Cas9 HNH domain remains trapped in the checkpoint intermediate, and the addition of Mg^2+^ facilitates the reorientation docking onto the target DNA [89].

### 6.1. Genetic Control for the Allosteric Modulation of Cas9-Targeting Specificity

#### 6.1.1. sgRNA

As sgRNA comprises a seed sequence and a non-seed sequence, the seed sequence comprises 10–12 bp adjacent to PAM that determines Cas9 specificity and is far more important than the rest of the gRNA sequence [11,37]. The sgRNA sequence plays a significant role in Cas9 specificity, which can be altered to improve it further. Most of these modifications improve Cas9 specificity when performed near the sgRNA 5′ end (i.e., the PAM distal end). Some more modifications include the extension of two extra guanine nucleotides [91], the truncation of two to three nucleotides [92], and partial DNA replacement [93]. In addition, some gRNA variants contain unnatural chemical modifications like 2′-O-methyl-3′-phosphonoacetate and bridged nucleic acids incorporated within the central part or near the 5′ ends of the gRNA targeting region [94]. The RNA-DNA heteroduplex formation can be interfered with by these diverse modifications at or near the PAM-distal end. These modifications further sensitize the Cas9 cleavage activity to mismatched DNA targets due to an enhanced threshold for crossing the conformational checkpoint.

#### 6.1.2. PAM

The activity of sgRNA and Cas9 also depends upon the sequence of PAM, as initial findings showed that NGG (where N is A, T, G, or C) represents the established sequence for PAM. However, some recent observations advocate that a type II CRISPR system may also use the PAM as NRG, where R represents A or G, despite having almost one-fifth the binding efficiency as compared to NGG. In the PAM sequence, the binding frequency of each base is different, as the first nucleotide is least conserved, with G having nearly 50% of the binding sites, while the second location has G in more than 90% of the binding sites [58,95]. It implies that NRG is not the optimal PAM sequence for DNA cleavage by Cas9 [96]. Every sgRNA has its own PAM, typically NGG, when designed via common CRISPR/Cas9 design tools like CRISPR design (http://crispr.mit.edu/ accessed on 18 January 2023), E-Crisp (www.e-crisp.org/E-CRISP/Designcrispr accessed on 18 January 2023), CRISPR design tool (http://www.broadinstitute.org/mpg/crispr_design/ accessed on 18 January 2023), Cas-OFFinder (http://www.rgenome.net accessed on 18 January 2023), or CROP-IT (http://www.adlilab.org/CROP-IT/homepage.html accessed on 18 January 2023). For the functional sgRNA, alternative NGGs may not exist if precise insertion or point mutations in the genome are adopted. However, the NRG (R = A or G) sequence can be considered an alternative, but with low cleavage efficiency [96].

### 6.2. Chemical Control to Regulate CRISPR/Cas9-Based Genome Editing

The chemical control approaches to regulate CRISPR/Cas9-based genome editing mainly include (i) the regulation of nuclease activity of Cas9 or different variants by small-molecule-triggered binding, (ii) the inhibition of Cas9 nuclease activity by anti-CRISPR proteins or degrons, and (iii) the scheme of bioresponsive delivery carriers to control the release of the CRISPR/Cas9 complex within specific cells and tissues [97].

#### 6.2.1. Control by Activators

Different inducible promoters in mammalian cells and animal models have been investigated as regulators to control Cas9 activity [98]. A doxycycline-inducible gRNA system has been developed that is responsible for Cas9-mediated genome regulation [99]. The doxycycline treatment promotes the robust induction of Cas9 and gRNA-mediated genome editing. However, these approaches show a slow response time and require additional factors, e.g., the reverse tetracycline transactivator [100]. In contrast, the methods that control protein synthesis by post-translational approaches offer better sequential resolution [101].

Cas9 enzyme activity has been disrupted by 4-hydroxytamoxifen (4-HT)-responsive intein inserted at specific sites of this protein [102]. The addition of 4-HT promotes the splicing of the intein and releases active Cas9. Even though the general activity of this engineered Cas9 was slightly inferior as compared to native Cas9, the ratio of on-target to off-target editing was almost sixfold higher. In another study, the hormone binding domain of the oestrogen receptor (ERT2) was fused with Cas9 to form iCas9, which enables firm temporal control of Cas9 using 4-HT [103]. In the absence of 4-HT, the ERT2 domain confiscates Cas9 in the cytoplasm, but upon the addition of this compound, the fusion protein swiftly translocates to the nucleus for genome editing. In addition, randomized insertional mutagenesis was performed to introduce a small domain into the Cas9 sequence for the screening of active variants to identify the structural hotspots within Cas9 that could tolerate additional protein domains [104]. Some insertion sequences include the ligand binding domain of the human oestrogen receptor-α at position 231 of Cas9 or dCas9, offering 4-HT-responsive Cas9 (arCas9) or dCas9 (darCas9) states.

Based on the chemically induced dimerization of split protein fragments, different small-molecule-controlled Cas9 systems have been developed. In this regard, rapamycin-mediated dimerization of FK506 binding protein 12 (FKBP) and the FKBP rapamycin binding domain (FRB) of the mammalian target of rapamycin (mTOR) was performed [105].

A split Cas9 was engineered, with the N-terminal fragment fused with the FRB domain and the C-terminal fragment fused to the FKBP domain [106]. Further, a nuclear localization signal (NLS) was added to the C-terminal fragment and a nuclear export signal (NES) was added to the N-terminal fragment, to avoid any unprompted reconstitution of the two fragments, thus minimizing basal activity in the absence of rapamycin. A low level of Cas9 activity was observed with this design, but irreversible activation was seen after the rapamycin addition. Furthermore, the induction of this split-Cas9 system with rapamycin led to substantial indels formation at the intended genomic loci with no significant off-target effects [106].

A reversible and dose-dependent transcriptional activation/repression by abscisic acid-inducible ABI-PYL1 and gibberellin-inducible GID1-GA1 heterodimerization domains was demonstrated in gene regulation [107]. In this situation, dCas9 was fused to either AGI or ABI, while the effector domains were fused to GID1 or PYL1, allowing multiplexed transcriptional regulation. After 24 h, a detectable rise in transcriptional activation was observed. Moreover, upon the removal of the inducer, such systems were reversible, with the activity approaching baseline in 4–5 days [108].

#### 6.2.2. Control by Inhibitors

There has been a surge of attention to the recent discovery of naturally occurring, genetically encoded CRISPR system antagonists termed anti-CRISPRs. These proteins have been planned to work as context-specific Cas9 inhibitors. Protein-based anti-CRISPRs are fewer than 200 amino acid polypeptides found in bacteriophage genomes used to inhibit the CRISPR/Cas9 system [109,110]. Such proteins help the phage dodge the bacterial immune response, thus helping phage propagation. Up until now, more than 20 different anti-CRISPR families have been characterized, targeting type I and type II CRISPR/Cas systems [111]. The anti-CRISPRs that target SpCas9 include AcrIIA2 and AcrIIA4 [112]. Different approaches have been found by which anti-CRISPRs interfere with the CRISPR/Cas system. These inhibitors can bind the gRNA-loaded CRISPR/Cas system, preventing its DNA binding [113]. Another approach involves binding to Cas effector proteins and blocking their recruitment for the activation of cascade complexes in the type I system [113] or directly inhibiting the nuclease activity in the Cas9 protein.

With the passage of expression time, the off-target activity of CRISPR-associated nuclease and DSB-induced toxicity increases. Thus, handicapping or controlled inhibition of Cas9 activity after a desired DSB would be expected to lessen these complications. A properly timed transfection of AcrIIA4 plasmid or protein can minimize the number of Cas9 off-target edits at VEGFA and HBB loci in K562 human cells [114].

The synthesis of small-molecule inhibitors for Cas9 is a challenging task due to multiple reasons. The identification of inhibitors requires very complex Cas9 assays, which are mostly unavailable. Being a single-turnover enzyme with substrates having picomolar affinity complicates the development of high-throughput assays. Some novel protein folds present in Cas9 limit the application of inhibitor design approaches [35]. The inactivation of two nuclease domains shuts down Cas9 activity completely. Furthermore, Cas9, being a DNA-binding protein, is often regarded as chemically intractable [115].

#### 6.2.3. Control by Degraders

The degradation of Cas9 is sometimes preferred over its inhibition, as there are some antibodies in humans against this protein [116]. Thus, Cas9’s specific immune response is a major hurdle in its development for therapeutic applications. A timely degradation of Cas9 in many scenarios is preferred over complete inhibition. Some post-translational regulation of proteins is performed by using small molecules [117,118]. The use of heterobifunctional molecules leads to the co-localization of target proteins, and specific ubiquitin ligases are used for the proteasomal degradation pathway [119,120]. The fusion of degrons against a specific protein induces degradation upon the addition of a small molecule.

The Cas9 protein has been linked with the FKBP12^F36V^ variant, which connects with specific E3/E2 ubiquitin ligases by the addition of heterobifunctional dTAG. This leads to ubiquitination and degradation of the whole fusion protein [121,122]. However, the direct fusion of degrons like dihydrofolate reductase (DHFR) or ER50 looks more convenient as compared to ubiquitinated degradation. DHFR is a destabilizing domain that rapidly targets fusion proteins for proteasome-mediated degradation. However, it can be stabilized by the addition of small-molecule inhibitors like trimethoprim (TMP) or 4-hydroxytamoxifen (4OHT). VEGFA gene editing by the Cas9-DHFR or Cas9-ER50 systems, treated with multiple concentrations of TMP or 4OHT, led to an enhanced on-target to off-target ratio [123]. The use of these small-molecule inhibitors is significant when CRISPR/Cas9 genome-editing is restricted to specific cells or a narrow window.

## 7. Stimuli-Responsive Nanoformulations of CRISPR/Cas9 for Their Targeted Delivery

Considering the differences between the tumor microenvironment (TME) and the environment of normal tissues, stimuli-responsive nanoformulations have been engineered to transport and release CRISPR/Cas9 components within the tumor cells. Generally, the nanoformulation is made responsive to internal stimuli, including hypoxia, pH, redox-reagents, ATP level, etc., and external stimuli, such as thermal, photo, magnetic, ultrasound, etc. [124]. The stimuli-responsive nanoformulations possess high therapeutic performance by minimizing the off-targeting risks of CRISPR/Cas9-based cancer management.

### 7.1. Internal Stimuli

#### 7.1.1. pH-Responsive Nanoformulations

Recently, pH-responsive nanocarriers have been widely used to transport and release the CRISPR/Cas9 system or its components within the TME. Cancer cells possess organelles with a low pH (pH 5.0–6.5) as compared to the pH value in the cytosol, blood, and normal tissue (~pH 7.4) [125]. Different types of materials, such as copolymers, inorganic molecules, and lipids, have been used to develop CRISPR/Cas9 pH-responsive nanocarriers for their efficient transport and controlled release within cancer cells. These nanocarriers possess pH-triggered disassembly properties. Nanoliposomes have been constructed with the thin film method using pH-responsive phospholipids, cationic phospholipids, cholesterol, and DSPE-PET2000 for effective CRISPR/Cas9 encapsulation up to 95% [126]. The decreasing pH led to an increased surface charge of this nanocarrier, and this pH sensitivity led to almost 80% cargo release within 20 h. The intratumoral injection of this CRISPR/Cas9 nanoformulation targeted to splicing HPV 16 E6/E7 in nude mice efficiently knocked out the system and significantly minimized tumor growth. In addition, this nanoformulation showed no biotoxin effect on any tissue architecture within normal organs [126].

**Figure 6:**
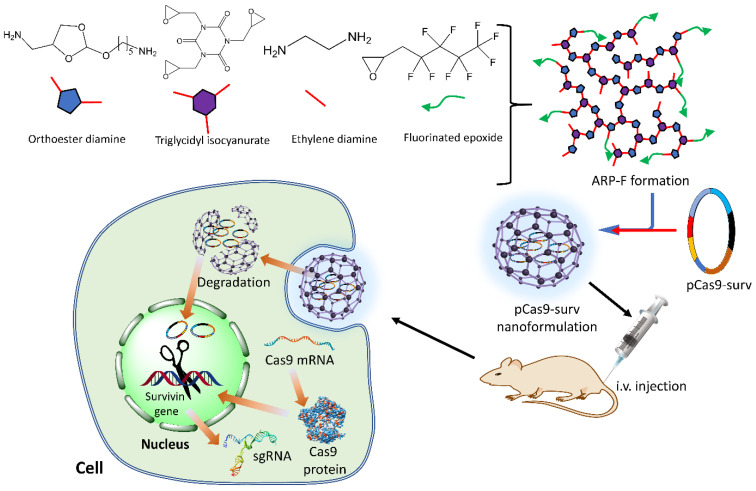
Molecular composition of ARP-F, its pCas9-suv nanoformulation preparation, and intracellular delivery strategy of CRISPR/Cas9 induced by pH response.

An acid-responsive polycation (ARP) nanoformulation was prepared through a one-pot ring-opening polymerization method. The engineered ARP possessed abundant ortho-ester linkages and hydroxy groups, with fluorinated alkyl chains forming the final structure [127]. A stable nanoformulation is generated through electrostatic interactions by condensing the CRISPR/Cas9 plasmid and ARP-F. In an acidic medium, the ortho ester linkages undergo degradation, releasing the loaded plasmid. This strategy has been reported for knocking out (KO) the survivin gene by pCRISPR/Cas9-surv, targeted by ARP-F nanoformulation, exhibiting tumor repression under both in vivo and in vitro conditions. Furthermore, the survivin gene KO led to enhanced sensitivity of cancer cells to anti-tumor drugs such as temozolomide, thus providing an efficient combination therapy for cancer treatment [127] (Figure 6).

**Figure 7:**
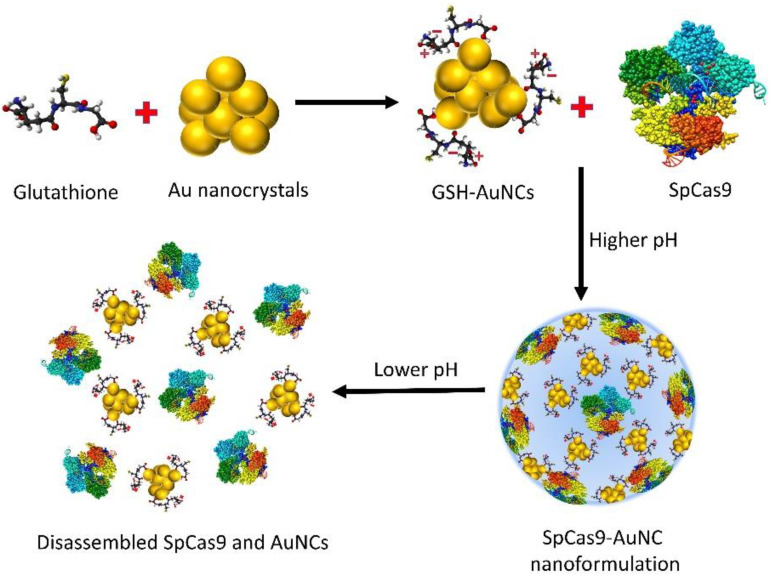
Diagrammatic representation of pH-induced assembly and disassembly of SpCas9-AuNCs. At higher pH, SpCas9 self-assembles with AuNCs through electrostatic interactions, whereas at lower pH, due to weak interactions between SpCas9 and AuNCs, disassembly of this nanoformulation occurs.

Some metal-containing self-assembled nanoformulations also show pH-responsive disassembly. SpCas9 has been reported to self-assemble through electrostatic interactions with gold nanoclusters (AuNCs) with carboxylic groups [128] (Figure 7).

Higher pH provides a stable environment for the assembly of SpCas9-AuNCs, while at lower pH, this composite efficiently disassembles owing to carboxylic group protonation in AuNCs, thus releasing SpCas9. A remarkable oncogenic E6 gene KO (34%), has been reported in cervical cancer cells by employing HPV 18 E6 sgRNA and SpCas9-AuNCs. In addition, no obvious KO effect has been observed by SpCas9-AuNCs in normal cells.

Cas9 RNP has been encapsulated with a zeolitic imidazole framework (ZIF-8) through self-assembly with Zn^2+^ and 2-methylimidazole linkers. The protonation capacity of imidazole linkers makes this system self-assembled, which is also pH-dependent. The data show that 60–70% of Cas9 was released at pH 5–6 within 10 min, while under physiological conditions, only <3% of Cas9 was released. The delivery of Cas9 RNPs led to a 3-fold reduction in enhanced green fluorescent protein (EGFP) gene expression and a 37% decrease in its fluorescence level [129].

In another study, the water-in-oil emulsion method was used to prepare pH-responsive silica-metal-organic framework hybrid NPs (SMOF). These NPs, with both ZIF-8 and silica, were found to incorporate hydrophilic payloads. The encapsulating efficiency of these NPs was reported to be more than 95% for CRISPR/Cas9 RNPs. However, SMOF presented a fast release of encapsulated RNPs because of the same pH-induced degradation. A subretinal injection of these NPs in murine retinal pigment epithelium (RPE) tissue exhibited enhanced genome-editing efficiency [130].

#### 7.1.2. GSH-Responsive Nanoformulations

The environment of sensitive reduction capacity around tumor sites has led to the idea of engineering nanoformulations with redox-responsive delivery approaches for CRISPR/Cas9 [131]. In recent years, glutathione (GSH)-triggered nanocarriers have been used as potential platforms for cancer cell genome editing. There exists a vast difference between intracellular GSH levels (2–10 mM) and extracellular GSH levels (2–10 μM) [132]. Recently, some GSH-responsive delivery platforms, such as bioreducible lipid NPs (LNPs) [133], phenylboronic acid-derived LNPs [134], and copolymers [135] loaded with CRISPR/Cas9, have been engineered for genome editing. The intracellular GSH easily cleaves the disulfide bond, and furthermore, this is also used to conjugate with some bioactive compounds [136].

**Figure 8:**
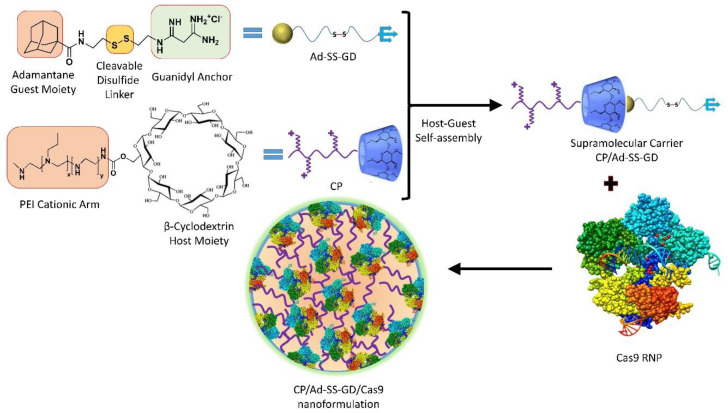
Diagrammatic representation of CP/Ad-SS-GD nanoformulation assembly for intracellular CRISPR/Cas9 delivery induced by GSH.

A supramolecular polymer has been engineered that performs a controlled release of Cas9-RNPs [137] (Figure 8). This supramolecular polymer system has been synthesized between disulfide-bride biguanide adamantine (Ad-SS-GD) and β-cyclodextrin-conjugate polyethylene-imine (CP) to generate CP/Ad-SS-GD. The RNPs loaded in such nanoformulations could be released up to 90% in 72 h in the presence of GSH, whereas in the absence of GSH, only 21.9% of RNP release has been reported. The RNP targeted nanoformulation against mutant Kirsten rat sarcoma virus (KRAS) and remarkably inhibited tumor development in both colorectal cancer xenograft models and colorectal cancer cells.

**Figure 9:**
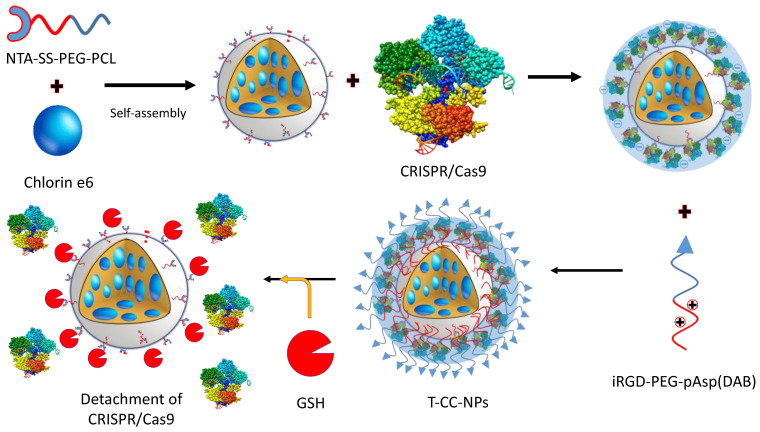
Diagrammatic illustration of reducing agent-sensitive nanoformulation containing Nrf2-targeting CRISPR/Cas9.

A combined tumor therapy with GSH-responsive micelles demonstrated that anticancer photosensitizer chlorin e6 (Ce6) and CRISPR/Cas9 RNP codelivery produce noticeable results [138] (Figure 9). These micelles were synthesized from nitrilotriacetic acid-disulfane-diyldipropionate-polyethyleneglycol-β-polycaprolactone (NTA-SS-PEG-PCL) and iRGD-PEG-β-polyaspartate-γ-1,4-butanediamine [internalizing RGD-PEG-pAsp-(DAB)]. The loaded RNP detachment occurred due to the disruption of the disulfide bond between PEG and NTA in response to GSH. This led to the disruption of the antioxidant regulator Nrf2 in both in vivo and in vitro conditions.

In CNE-2 xenograft mice, this led to improved tumor sensitivity to NIR/Ce9-generated ROS. This study also supports the synergism between photodynamic therapy and CRISPR/Cas9-based genome editing.

A polymer-based, GSH-responsive nanoformulation was engineered for the delivery of CRISPR/Cas9 [133]. In this nanoformulation, azide-conjugated β-cyclodextrin was covalently cross-linked with DBCO-modified branched DNA types (7F or 7R), a linker, and RNP was assembled to synthesize the nanoformulation. When incubated with the GSH, the antisense and RNPs were easily released. The Michigan Cancer Foundation's (MCF) human breast cancer cells demonstrated significant inhibition of cell proliferation by targeting polo-like kinase 1 (PLK1) through this nanoformulation. The tumor-bearing mice also showed suppressed tumor growth by using this approach.

For the treatment of tumors, another GSH-responsive nanoformulation has been engineered by using an EZH2-targeted CRISPR/Cas9 plasmid in a platinum-backed polymeric NP [139]. The internalization of this nanoformulation led to its breakdown due to the transformation of Pt(IV) to Pt(II) induced by GSH. The plasmid DNA was dispersed in the cytoplasm, leading to EZH2 knockout effectively under in vivo (21.3%) and in vitro (32.2%) conditions. The EZH2 suppression promotes the downregulation of H3K27me3, possibly increasing the accessibility of Pt(II) to nuclear DNA and enhancing apoptosis. A significant amount of growth inhibition has been reported against subcutaneous xenograft tumors using this approach.

### 7.2. External Stimuli

In the recent past, the control of CRISPR/Cas9-mediated genome editing by external physical control has become a popular strategy due to its high precision and non-invasiveness [140,141]. Innovative CRISPR platforms have been constructed by engineering physically responsive elements that are light-, magnetic-, heat-, and ultrasound-responsive. Once the stimulation through different physical factors approaches, the activity, structure, function, transport, expression, and release of the CRISPR/Cas9 system are controlled. The following sections describe four external physical factors that control the activity of CRISPR/Cas9 genome editing (Table 3).

#### 7.2.1. Photo-Responsive Targeting of CRISPR/Cas9

Several photoresponsive molecules have been constructed in the past decade to optically control the genome-editing activity of the CRISPR/Cas system [142,143]. Some photosensitive molecules, like spiropyran derivatives and azobenzene derivatives that contain *o*-nitrobenzyl moieties, readily undergo photoisomerization or ester bond cleavage when exposed to light [144,145]. Different light-controlled CRISPR/Cas9-based genome engineering techniques have been designed by exploiting such features of these molecules. An optogenetic two-hybrid system was created from two independent components: a genomic anchor (dCas9) fused with the photo-sensitive cryptochrome-interacting (CIB1) protein to form the dCas9-CIB1 complex, and a cryptochrome circadian clock 2 (CRY2) fused with a separate effector domain to form the CRY2-activator complex [146,147,148]. After the activation with blue light (~450 nm), the CIB1-effector complex was employed to form a biopolymer, the dCas9-CIB1-CYR2-effector complex, that broadened the activation functionality of Cas9 [148,149]. This phenomenon is reversed when the cells are incubated in the dark [150].

Light has also been used to change the Cas9 nuclease activity as a split Cas9 (paCas9) with N and C terminal domains fused to light-inducible dimerization domains (pMag and nMag) [133,148]. In the absence of light, each split fragment of Cas9 is inactive, while blue light promotes heterodimerization of split Cas9 fragments through pMag-nMag interactions, thus restoring Cas9 activity [148,151]. The paCas9 can be used in genome editing and modifications like a wild-type Cas9. In parallel, a different photo-switchable Cas9 was engineered (psCas9), having a single polypeptide chain [152]. The PAM-interacting (PI) and REC2 domains of psCas9 were introduced by the photo-dissociable dimeric fluorescent protein (pdDronpa1) [153]. The enclosed pdDronpa domains homodimerize and sterically inhibit psCas9 activity without light activation at 500 nm. However, the illumination with the same wavelength light caused the pdDronpa1 to dissociate, which resulted in the restoration of Cas9 activity [152,153].

Furthermore, the Cas9-RsLOV2 monomer has been constructed by fusing Cas9 and the *R. sphaeroides* light-oxygen-voltage (LOV) domain (RsLOV) [154]. In the absence of light, the two Cas9-RsLOV2 monomers homodimerize, imposing a steric inhibition of Cas9 activity. However, a blue light shock promotes Cas9-RsLOV2 dimer dissociation and reversion to Cas9-RsLOV monomer, which has high targeting and nuclease activity [154]. A photocaged lysine was inserted at the specific domain of Cas9 responsible for the binding of sgRNA, thus rendering it inactive [155]. The exposure to UV light for 120 s leads to the removal of photocaged lysine, leading to the restoration of Cas9 activity.

The light activation has also been used to change the sgRNA activity using a photocleavable ssDNA oligonucleotide (termed a protector) that binds with the target region of sgRNA [156]. The hybridization of a protector with sgRNA leads to the inhibition of sgRNA:DNA base pairing, which occurs until the ssDNA oligonucleotide is photolyzed by UV irradiation. It releases sgRNA from it to bind the target DNA again for subsequent genome editing; however, this method is irreversible.

**Figure 10:**
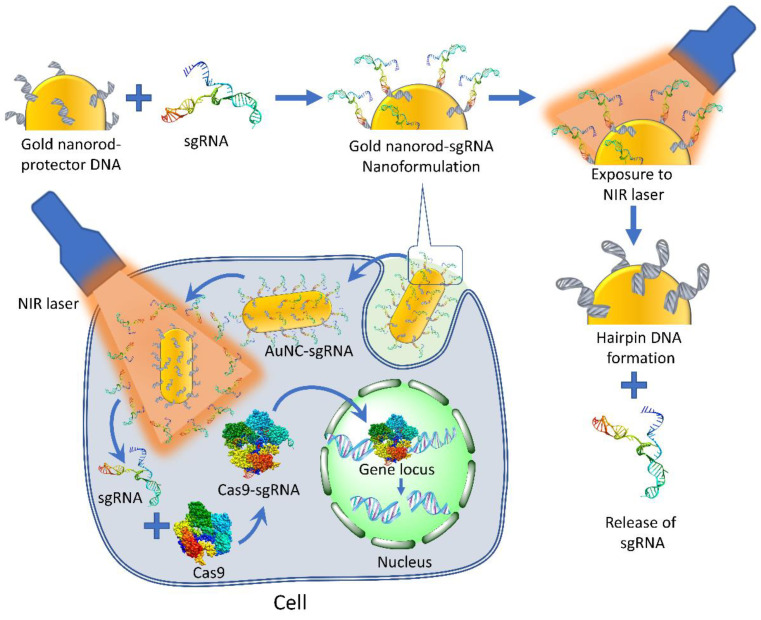
Photo-responsive intracellular CRISPR/Cas9 nanoformulation delivery. sgRNA hybridization with a protector DNA, sgRNA release after heat activation, hairpin structure formation of protector DNA, and sgRNA release within the cells.

Peng et al. designed an experiment for the delivery of sgRNA using gold nanorods modified with a protector DNA strand that hybridizes with sgRNA. These engineered gold nanorods were delivered into A549-GFP/Cas9 cells expressing Cas9 and GFP proteins. After the irradiation with near-infrared (NIR) at 808 nm, the produced heat dehybridized the protective DNA/sgRNA complex to release the sgRNA. The remaining protector DNA instantly formed a hairpin structure on the gold nanorod surface that prevented rehybridization of the released sgRNA with DNA. The released sgRNA was accordingly bound to Cas9. sgRNA bound to Cas9 was expressed in pre-transfected cancer cells for gene editing [157] (Figure 10).

#### 7.2.2. Heat-Responsive Targeting of CRISPR/Cas9

The approach of remote switching of gene expression regulation by heat shock has been applied for the regulation of Cas9 activity. Lipid-encapsulated thermosensitive gold nanoparticles (AuNPs) have been engineered to carry the CRISPR/Cas9 release system [158]. In this method, nucleus-targeting TAT peptides and cations were joined to AuNPs to construct cationic AuNPs. By electrostatic interaction, the negatively charged Cas9-spPlk-1 plasmid (CP) was condensed on these cationic AuNPs to form a complex AuNPs/CP (ACP), which was further coated by lipids to make lipid-encapsulated ACP (LACP). Through photothermal effects, the localized surface plasmon resonance (LSPR) AuNPs can generate heat. The AuNPs in LACP could localize the heat source and trigger TAT/CP release from the AuNPs through photothermal effects from 514 nm laser irradiation. The released TAT/CP complex transferred to the nucleus, and the targeted gene (Plk-1) was potentially knocked down, which inhibited the tumors under in vivo conditions [158].

A semiconductor polymer brush (SPPF) was designed as a photo-thermally triggered CRISPR/Cas9 release system with NIR-II photothermal transducer and imaging characteristics [159]. In comparison to the LACP system, this system adds one functionality of NIR-II imaging: tracking the distribution of gene editing tools within the body by using remote photothermal triggers in real-time [141]. At the target tissue or cell, the NPs generate heat through laser-irradiated (808 nm) photothermal effects. It facilitates the endolysosomal escape of NPs and releases CRISPR/Cas9 and dexamethasone payloads. Dexamethasone helps in the transport of the CRISPR/Cas9 system into the nucleus for target genome editing [159]. Furthermore, thermal shock can also activate heat shock promoters of CRISPR/Cas9 that result in conditional genome editing in various cell types at various developmental stages [141].

#### 7.2.3. Ultrasound-Responsive Targeting of CRISPR/Cas9

The increasing applications of ultrasound have played a similar role to that of light and heat in releasing payloads from different targeted nanocarriers [160,161]. The nanomotors driven by ultrasound rapidly penetrate plasma membranes and provide acoustic activity within the intracellular spaces, thus acting as efficient vehicles to conduct intracellular drug delivery [162]. Recently, nanomotors propelled by ultrasound activation were used as carriers to quickly and directly deliver the Cas9/sgRNA complex within cells [160]. The gold nanowire (AuNW) surface has been connected to the Cas9/sgRNA expression plasmid through disulfide bonds to form Cas9/sgRNA-AuNW complexes. The ultrasound activation of these complexes leads to active movement and promotes their internalization into the cytoplasm.

Microbubble-conjugated nanoliposomes (MB-NLs) have been used as carriers of the Cas9/sgRNA complex. These MB-NLs containing the Cas9/sgRNA complex can significantly deliver their shipments to specific target sites under ultrasound activation [161]. Furthermore, the Cas9/sgRNA delivery has been achieved within dermal papilla cells (DPC) with the help of high acoustic wave ultrasound with a frequency of 1–5 MHz [161].

#### 7.2.4. Magnetic Field-Responsive Targeting of CRISPR/Cas9

It has been reported that under in vitro and in vivo conditions, the molecular or cellular behavior of magnetic nanomaterials changes with the stimulation of an external magnetic field [163,164]. Some magnetic nanomaterials have been used to construct CRISPR/Cas9 system carriers for on-demand and on-target delivery within target cells or tissues [165]. A magnetically guided nanoformulation was engineered between the Cas9/sgRNA complex and magneto-electric NPs (MENPs) [165]. These Cas9/sgRNA-MENPs can cross the blood-brain barrier (BBB) under the influence of a magnetic field to edit the HIV gene to reduce HIV infection in microglial cells [165]. These carriers are ferromagnetic, 25 ± 5 nm in size, nontoxic up to 50 μg, and can cross the BBB under a static magnetic field. Upon stimulation with an external ac-magnetic field, these MENPs cause polarization changes on their surface, resulting in the bond breakdown between MENPs and Cas/sgRNA. It leads to the on-demand release of Cas9/sgRNA in target cells and performs the gene knockout and other mutations [165].

Magnetic fields have also been used in recombinant magnetic NPs baculoviral vectors (MNP-BV-CRISPR) for performing CRISPR/Cas9-mediated genome editing at some specific sites [166]. Upon exposure to a magnetic field, these NPs disperse in aqueous buffers and migrate against the field gradient as nanomagnets [166]. In addition, these MNP-BV-CRISPR NPs can be inactivated by the complement system of serum, thus behaving as off-switches of gene editing. The external magnetic field acts as an on-switch by locally controlling the margination and cell entry of the MNP-BV-CRISPR NPs to control gene-specific editing [166]. The magnetic stimulation of MNP-BV-CRISPR and MENPs-Cas9/sgRNA organizes the in vivo spatiotemporal regulation of CRISPR gene editing. However, this regulation system has not been fully studied yet.

**Table 3:** Examples of genetic regulation, chemical control, and physical control approaches for CRISPR gene editing.

Control Type	Edited Gene	Cell or Organism Models	Key Molecule/Structure	Reference
**Genetic regulation approaches of CRISPR/Cas9 gene editing**				
Modular CRISPR fusion system	human ASCL1, ZFP42 and OCT4 genes, adipogenic genes, IL1RN, GFP reporter gene, pluripotency gene NANOG	HEK-293T, MSC, yeast cell, hPSC, HeLa cells	copies of VP16, VP64, p65, multiple sgRNAs, AcrIIA1-4, VP64-p65- Rta tripartite, KRAB, SID4X	[97,167]
Cell-specific promoter	mouse CD2 gene, hepatitis B virus (HBV) genome, zebrafish urod gene, macrophage-specific gene sgNtn 1, LON-2, C. elegans somatic cell DPY-5, and GFP gene	HEK-293T cell, HepG2.2.15 cell, Huh7 cell, mouse T cell, B cell, macrophage, spleen cell, neutrophil, monocyte, zebrafish, C. elegans	Macrophage-specific promoter, liver-specific promoter, erythrocyte-specific gata1 promoter, CD4 promoter, Egg cell-specific promoter	[168,169]
**Chemical approach to control CRISPR gene editing**				
Small molecule activators	SOX2 gene, GFP reporter gene, PPP1R12C, EMX1, VEGFA, ASCL	mouse zygote, HEK-293T cell, HEK293-GFP cell, STF3A cell line, N2A cell	mouse serum PCSK9 gene, GFP reporter gene,	[106,170]
Small molecule inhibitors	*E. coli* genome, CD71, and CXCR4 genes, VEGFA, endogenous IL1RN or NANOG gene, GFP or BFP reporter gene	HeLa cell, hPSC, K562 cell, *E. coli*, U2OS cell, HEK-293T cell, NIH/3T3 cell	ubiquitin ligase, AcrIIA1-4, DHFR and ER50, unstable protein domains	[171,172]
Bioresponsive delivery carrier	mouse serum PCSK9 gene, GFP reporter gene	mouse liver, mouse hepatocytes, HEK-293 T cell, lung and spleen tissues	lipid molecules with different charges, bioreducible BAMEA-O16B lipid NP	[173,174]
**Physical approach to control CRISPR gene editing**				
Light	human GRIN2B gene, zebrafish ASCL1a and HSP70 gene, the promoter of the human ASCL1 and IL1RN genes, CD71 gene, mCherry reporter gene	ZF4 cell, HEK-293T cell, *E. coli*, HeLa cell	dimeric fluorescent protein pdDronpa1, photo-cleavable ssDNA oligonucleotide, pMag-nMag, CIB1-CYR2- effector, photo-caged lysine, Cas9- RsLOV2 monomers	[175,176]
Heat	GFP reporter gene, Plk-l gene, LON-2, C. elegans somatic cell DPY-5, and GFP gene	HEK-293T cell, HCT 116 cell, A375 cell, C. elegans	APC, AuNPs, Phsp, SPPF-Dex nanoparticles	[158,159]
Magnetic field	mouse VEGFR2 gene, HIV LTR gene,	Hepa 1-6 cells, microglial (hμglia)/HIV (HC69) cells	magnetic iron oxide nanoparticles (MNP-BV), magneto-electric nanoparticles (MENPs)	[165,166]
Ultrasound	steroid type II 5-alphareductase gene, GFP reporter gene	DPC cell, androgenic alopecia, B16F10 cell, mouse	microbubble conjugated nanoliposome (MB-NL), gold nanowires (AuNWs),	[160,161]

Abbreviations: AcrIIA1-4: anti-CRISPR-associated protein; APC: a cationic polymer-coated Au nanorod; AuNPs: lipid-encapsulated gold nanoparticles; CD71 and CXCR4: cell surface transmembrane protein genes; DPC cell: dermal papilla cell; GFP: green fluorescent protein; HEK-293T cell: human embryonic kidney 293T cell; hPSC: human pluripotent stem cell; Huh7 cell: human liver cancer cell line; K562 cell: human K562 erythroleukemia cell; KRAB, SID4X, MXl1, and WRPW: transcription repression domain; MSC: mesenchymal stem cell N2A cell: mouse neuroblastoma N2a cells; NIH/3T3: mouse embryonic cells; Phsp: heat-shock-inducible promoter; Plk-l gene: gene for regulator of mitosis; SPPF-Dex: NPs consist of alkyl side chains, dexamethasone (Dex), fluorinated polyethylenimine (PF), and PEG chains; STF3A: a cell that carries a Wnt-responsive luciferase reporter and also strongly expresses a Wnt ligand; VP64, p65, VP16, and Rta: transcription activation domains; ZF4 cell: zebrafish embryonic fibroblast.

## 8. Difference between Genetic, Chemical, and Physical Control for CRISPR/Cas9 On-Target Strategies and Their Limitations

A firm lack of precision severely constrains the full potential applications of CRISPR/Cas9-based genome editing in biological systems. However, some gene editing tools have clear-cut genetic, chemical, or physical control that can be rapidly and reversibly programmed to target specific loci [175]. Different transcription effectors or cell-specific promoters have been engineered to control the transcription or gene editing of interested sites. The addition of numerous activation domains like VP16 (such as dCas9-VP48, dCas9-VP96, and dCas9-VP192) improves the transcriptional activation efficiency of target genes. The specific promoters facilitate Cas9 expression only in specific cells without involving some other cells [176].

The utilization of chemical approaches mainly controls the start, duration, intensity, and spot of genome editing by CRISPR/Cas9 by using small molecule activators, inhibitors, or bioresponsive delivery carriers [122,177]. For example, the use of an anti-CRISPR polypeptide prevents Cas9/sgRNA binding to target DNA, while oestrogen receptor (ERT2) fusion grips Cas9 out of the cellular nuclei. The use of inteins within Cas9 triggers its nuclease inactivation, which can be restored by using 4-HT. The destabilized domain DHFR or ER50 promotes proteasomal degradation of Cas9. The use of selective organ-targeting NPs (SORT) performs targeted delivery of mRNA and CRISPR/Cas9-based genome editing in different organs like the spleen, lung, and liver [160]. Some small-molecule inhibitors or activators can regulate CRISPR-based genome editing by time-based mechanisms to control the onset, duration, and intensity of CRISPR gene editing, with weaker control over the spatial dimension. However, bioresponsive delivery carriers and cell-specific promoters can achieve enhanced control of CRISPR genome editing in the spatial dimension [178].

In comparison to genetic regulation, the use of different chemicals and physical approaches has time and space control for CRISPR/Cas9 gene editing by using a platform of different delivery carriers such as temperature-dependent, photo-responsive, magnetic field-related, or ultrasound-responsive strategies [179]. For example, a heat shock response is important for the heat shock promoter (Phsp) for CRISPR/Cas9 cassettes as it permits restricted gene editing of some target cells at various developmental stages. In addition, the pdDronpa1 domain regulates Cas9 activity through spatiotemporal control by light irradiation [121,180].

The ultrasound activation controlled by the sonoactive gold nanowires (AuNWs) promotes Cas9/sgRNA internalization within the cytoplasm of specific cells. The release of Cas9/sgRNA on-demand in target tissues was performed by using MENPs that induce polarization changes on the surface after the stimulation with an external ac-magnetic field. Thus, physically remote-controlled on/off CRISPR gene editing is performed in real-time, enabling good spatiotemporal specificity, non-invasiveness, and easy tenability as compared to chemical strategies [160].

## 9. Critical Constraints of CRISPR/Cas9 System to Clinical Translation: Future Prospects and Challenges

The development of physico-chemical control over CRISPR/Cas9-based genome editing by the collaboration between biomaterial researchers and biological scientists has greatly helped in understanding genome engineering. However, several limitations still exist that limit its extensive purpose in clinical practice for different disease management. For example, gene regulation requires a cell-specific promoter that cannot be generalized. Thus, highly active and more effective cell-specific promoters need to be screened for this concern [181]. In parallel, several critical barriers exist to applying the full potential of chemical strategies for clinical translation.

Some chemical approaches require the least tuning; for example, additional factors are often required for small-molecule control strategies within target cells. This makes the process difficult to implement for clinical translation. Furthermore, a split Cas9 architecture consists of two fragments that consequently require rapamycin-binding dimerization domains to restore full activity. This makes the method inconvenient to implement during full potential clinical translation because multiple Cas9 fragments are required [88,91]. The burden of increased toxicity due to some chemicals like doxycycline and rapamycin also affects their implementation in clinical applications. The background activity of some small-molecule-activated systems hampers the precise control of the CRISPR genome editing strategy, thus making it difficult for clinical applications [182,183].

The application of physical strategies for CRISPR/Cas9 genome editing for clinical translation also faces some critical barriers. The deep penetration of light inside deeper biological tissues is hindered due to the highly complex biomolecules that hinder light-mediated CRISPR/Cas9-based clinical translation. Besides this, light and its deep tissue penetration also show some phototoxic effects, thus limiting its implementation in clinical applications [184].

In many cases, photothermal effects are the best choice as a heat source; however, the lower photothermal conversion efficiency of some carriers can hamper their heat activation. The heat shock promoters having high activity in some target cells or tissues can also be insufficient. The magnetic and ultrasonic-controlled materials can overcome some depth limitations of light-driven strategies and thus seem to be more innovative for clinical settings; however, more work needs to be done in the near future to explore such materials [185].

Even though CRISPR/Cas9 has proved to be a promising therapeutic approach for diverse cancers, this genome-editing system still suffers from several limitations that make it challenging to be used in clinical trials. Some of the challenges to overcome include off-targeting, immunogenicity, delivery methods, polymorphism, and ethics. These are some of the major challenges for CRISPR/Cas9 that need to be sorted out in the near future. The strategies to overcome the main challenges of the use of CRISPR/Cas9 have been elaborated somewhere else [186].

## 10. Conclusions

The progress in the characterization of the CRISPR/Cas9 system up to a sophisticated genome-editing level has led to a great transformation in life science. It has enabled many advances in basic research and established a promising foundation for the advancement of human therapeutics, including cancer management. However, major challenges remain for CRISPR/Cas9 clinical applications. Different variants of Cas9, including those with paired nickase, point mutations, fused miCas9, chimeric dCas9-FokI, and others, have been synthesized and possess high fidelity. These engineered Cas9 variants maintain cleavage activity and possess reduced off-target effects to different extents. Thus, selecting a suitable Cas9 endonuclease variant is highly significant but challenging. Even though a few high-fidelity Cas9 variants have been shortlisted, they are still far from perfection, as some variants are generated at the cost of cleavage activity loss. Some more challenges related to CRISPR/Cas9 applications include their safe and efficient delivery at in vivo target sites. However, nanotechnology-based stimuli-responsive delivery of CRISPR/Cas9 for cancer genome editing flags a new way for its clinical translation. Some difficult barriers to this endonuclease system's delivery in vivo have been sorted out by encapsulation, target delivery, controlled release, cellular internalization, and endosomal escape. Although all the requirements of the clinical trials are not entirely fulfilled by most of the currently used CRISPR/Cas9 nanocarriers, the perspectives are certainly positive. The future advances in nanotechnology-based pinpoint CRISPR/Cas9 targeting strategies at tumor sites will be scaled up for cancer therapeutics applications.

## Figures and Tables

**Table 1:** Summary of SpCas9 variants with mutation locations studied in different cell lines.

Cas9 Variants	Mutations Location/s	Year of First Introduction	Cell Type and Reference
SpCas9 nickase	D10A or H840A	2013	HEK293FT cells [57], HUES62 hES cells [57], human embryonic stem cells [58]
dCas9-FokI	D10A, H840A	2014	HEK293 cells [59], U2OS cells [59], HUES9 cells [60]
SpCas9-D1135E	D1135E	2015	U2OS cells [21]
eSpCas9	K810A, K1003A, R1060A	2016	HEK293 and HEK293T cells [16,61,62]
SpCas9-HF1	N497A, R661A, Q695A, Q926A	2016	U2OS cells [15], HEK293T cells [61,62]
HypaCas9	N692A, M694A, Q695A, H698A	2017	U2OS cells [18], HEK293T cells [61,62], mouse zygotes [63]
HiFiCas9	R691A	2018	human hematopoietic stem and progenitor cells [64], primary T cells [64]
xCas9	E108G, S217A, A262T, S409I, E480K, E543D, M694I, E1219V	2018	*E. coli* cells [23], HEK293T cells [62]
Sniper-Cas9	F539S, M763I, K890N	2018	*E. coli* cells [19], HEK293T cells [61], HeLa cells [19]
evoCas9	M495V, Y515N, K526E, R661Q	2018	Yeast cells [17], 293multiEGFP, 293blastEGFP, and HEK293T cells [61]
SpartaCas	D23A, T67L, Y128V, D1251G	2020	T cells [65]
LZ3Cas9	N690C, T769I, G915M, N980K	2020	HEK293T cells [61], K562 cells [61], U2OS cells [61]
miCas9	No mutation, SV40 NLS linker fused with brex27 motif	2020	Induced pluripotent stem cells [66], airway epithelial cells [66], fibroblast cells [66]
SuperFi-Cas9	Y1010D, Y1013D, Y1016D, V1018D, R1019D, Q1027D, K1031D	2022	HEK293 cells [67], neuro-2a mouse neuroblastoma cells [67]

**Table 2:** Different Cas9 variants and their salient features.

Cas Variants	Mechanisms of Action	Average Indel Frequency	Advantages and Limitations	Reference
SpCas9 nickase	Use dual-RNAs for site-specific DNA cleavage	75 and 60%	● Enhanced target specificity. ○ Rational design of sgRNAs on the plus and minus strands within a limited distance.	[80]
eSpCas9	Positively charged residues neutralization within the non-target strand, which thereafter weakened non-target strand binding and encouraged rehybridization between the non-target and target DNA strands	40%	● Decrease the off-target activities and maintain efficient on-target editing. ○ The unknown target discrimination and fidelity mechanism needs to be further improved.	[16]
SpCas9-HF1	Reduce the cleavage rate of DNA but have no effect on DNA reversion and release rate	34%	● Comparable to wild-type SpCas9, a high-fidelity variant retains on-target activities with >85% of sgRNAs. ○ The unclear mechanism of target discrimination and fidelity needs to be further improved.	[15]
HypaCas9	In wild-type SpCas9, a quadruple substitution in the REC3 domain	30%	Without affecting the on-target genome editing, possess higher genome-wide fidelity.	[18]
HiFiCas9	Between RNP and its substrate DNA, the weakening of non-specific interactions	Similar to WT Cas9	Diminished off-target effect and retained WT level on-target activity.	[64]
xCas9	Closing PAM or the DNA-sgRNA interface refines the DNA-RNA contact region	32%	● Improve the target specificity and extend the target range; present lower off-target activity and higher DNA specificity. ○ Profoundly diminished xCas9 activity at target sites with NGH PAM.	[22]
Sniper- Cas9	Weakening of non-specific interactions between RNP and its DNA substrate	46%	Retain WT-level on-target activity with diminished off-target effect.	[19]
evoCas9	Weakening non-specific interactions between RNP and its substrate DNA	15%	Retain WT level on-target activity with diminished off-target effect.	[17]

## Data Availability

Not applicable.

## Abbreviations

4-HT: 4-hydroxytamoxifen; ARP: acid responsive polycation; ATF: artificial transcription factor; AuNCs: gold nanocrystals; CRISPR/Cas9: clustered regularly interspaced short palindromic repeats-associated protein 9; crRNA: CRISPR RNA; DHFR: Dihydrofolate reductase; DSB: double stranded break; EGFP: Enhanced green fluorescent protein; EMT: epithelial to mesenchymal transition; FRET: Forster resonance energy transfer; gRNA: guide RNA; GSH: glutathione; HDR: homologous directed repair; HIV: human immunodeficiency virus; KO: knock-out; mTOR: mammalian target of rapamycin; NHEJ: nonhomologous end joining; NLS: nuclear localization signal; NPs: Nanoparticles; NUC: nuclease; PAM: protospacer adjacent motif; PDB: protein data bank; REC: recognition; RPE: retinal pigment epithelium; SAD: single wavelength anomalous dispersion; sgRNA: single guide RNA; TME: tumor microenvironment; TMP: trimethoprim; tracrRNA: trans-activating RNA; UCSF: University of California, San Francisco; WT: wild type.
